# Multi-Slice Computed Tomography Analysis in Patients Undergoing Transcatheter Aortic Valve Replacement – Impact of Workflows on Measurement of Virtual Aortic Annulus and Valve Size

**DOI:** 10.3389/fcvm.2022.877511

**Published:** 2022-06-21

**Authors:** Kerstin Piayda, Katharina Hellhammer, Verena Veulemans, Shazia Afzal, Kathrin Klein, Nora Berisha, Pia Leuders, Ralf Erkens, Julian Kirchner, Houtan Heidari, Malte Kelm, Gerald Antoch, Tobias Zeus, Christine Quast

**Affiliations:** ^1^Department of Cardiology, Pulmonology and Vascular Medicine, Heinrich-Heine-University Düsseldorf, Düsseldorf, Germany; ^2^Department of Diagnostic and Interventional Radiology, Heinrich-Heine-University Düsseldorf, Düsseldorf, Germany; ^3^CARID (Cardiovascular Research Institute Düsseldorf), Düsseldorf, Germany

**Keywords:** TAVI, TAVR, MSCT, valve sizing, outcome assessment

## Abstract

Exact and reliable measurements of anatomical dimensions in pre-procedural multi-slice computed tomography (MSCT) scans are crucial for optimal valve sizing and clinical results of transcatheter aortic valve replacement (TAVR). This study aimed to investigate interrater reliability between routinely used workflows for pre-procedural analysis. MSCT scans of 329 patients scheduled for TAVR were analyzed using both a 3mensio and SECTRA IDS7 platform. The results were retrospectively compared using the intraclass correlation coefficient, revealing excellent correlation in the analysis of simple diameters and poor correlation in the assessment of more complex structures with impact on calculated valve size.

## Introduction

Since its introduction in 2002, transcatheter aortic valve replacement (TAVR) has evolved as an inherent part of cardiovascular care delivery. Over recent years, the implantation technique and pre-procedural assessment advanced tremendously to ensure ideal prosthesis placement and fitting. Especially, multi-slice computed tomography (MSCT) scans have been deeply integrated into daily clinical practice to guarantee optimal valve sizing and clinical results. MSCT scans may be evaluated by different analysis platforms, workflows, and specialties influencing clinical routine and analysis of anatomical dimensions.

We, therefore, investigated the interrater reliability of workflows routinely used by radiologists and cardiologists in the analysis of relevant anatomical dimensions in pre-procedural MSCT scans of patients undergoing TAVR.

## Methods

Three hundred twenty-nine patients with severe, symptomatic aortic stenosis, and scheduled for TAVR underwent non-enhanced, contrast-enhanced, electrocardiogram-gated, and high-resolution MSCT (150 ms, 128 × 0.6 mm, “SOMATOM Definition AS+”, Siemens Healthcare) for pre-procedural planning from September 2015 to January 2018. The best systolic phase was used to reconstruct axial images with a slice thickness of 0.6–1 mm, and measurements were performed in accordance with best practice recommendations (1). Each data set of MSCT images was transferred to a dedicated workstation (3mensio Structural Heart^TM^, Pie Medical Imaging BV, Maastricht, The Netherlands) for evaluation by independent cardiologists (Table 1, named “examiner”). In case of complex anatomy or difficult image quality, a dedicated cardiological expert re-evaluated the measurements of the cardiological examiner (Table 1, named “Expert”). During this period, this was done in 20% of patients and resulted in high inter-operator reproducibility. Data were directly analyzed with a PACS system workstation (SECTRA IDS7, Sectra AB, Linköping, Sweden) for relevant anatomical structures by a specialized radiologist. Both specialties were extensively trained with internal validation in their routinely used workflow, and workflow users were blinded to the results of the other workflow. All measurements were retrospectively compared using the intraclass correlation coefficient (ICC, Pearson correlation with two-way random/absolute agreement model). TAVR has been carried out based on the 3mensio system, which represents the reference for measurements. During this period, the size of the implanted valves was strictly chosen according to the best practice recommendations of the manufacturers, which are indicated in the respective sizing charts of Edwards (Sapien 3) or Medtronic (Evolut R and Evolut Pro).

**Table 1:** Computed tomography (CT) evaluation and interclass correlation between 3mensio and Sectra IDS7.

	**3mensio**		**Sectra IDS7**	**ICC 95% CI**
	**Examiner**	**Expert**		
Virtual aortic annulus (mm)	22.9 ± 2.0	23.6 ± 2.2	24.7 ± 3.0	0.462 [0.17–0.63]
Sinotubular junction (mm)	27.3 ± 3.5	28.0 ± 3.4	26.8 ± 3.6	0.762 [0.70–0.80]
Sinus of valsalva (mm)	31.3 ± 3.8	32.0 ± 3.9	32.7 ± 3.8	0.627 [0.47–0.72]
Aorta ascendens diameter (mm)	31.9 ± 4.2	31.5 ± 3.8	31.3 ± 3.7	0.756 [0.69–0.80]
Distance to left coronary artery (mm)	13.4 ± 2.6	13.5 ± 2.2	12.3 ± 2.9	0.563 [0.41–0.67]
Distance to right coronary artery (mm)	14.6 ± 3.9	14.1 ± 3.6	13.6 ± 3.5	0.594 [0.46–0.68]
Left ventricular outflow tract angle (degree)	60.2 ± 6.2	58.0 ± 5.9	55.9 ± 15.2	0.025 [0.18–0.28]

**Figure 1:**
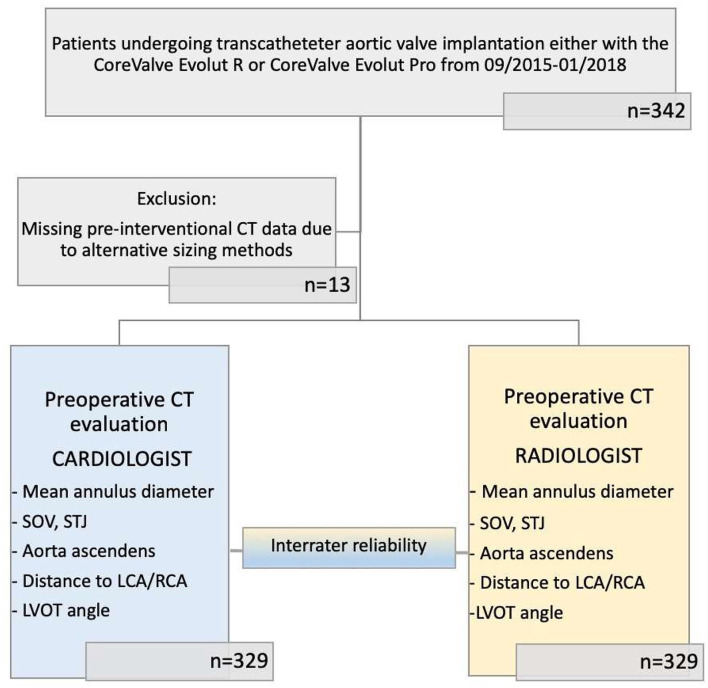
Modified CONSORT flow diagram. Cohort for CT evaluation comprises 329 patients with severe aortic valve stenosis scheduled for TAVR. Each patient has been evaluated by both 3mensio by cardiologists and Sectra IDS7 by radiologists.

The study design and patient selection process are illustrated in Figure 1. The study was approved by the local ethics committee, performed in accordance with the Declaration of Helsinki, and registered at Clinical Trials (NCT01805739).

## Results

The interrater reliability ranged from excellent in the prediction of simple two-dimensional distance measurements like the sinotubular junction (3mensio: 27.3 mm ± 3.5 vs. Sectra IDS7 26.8 mm ± 3.6, ICC.762 [0.70–0.80]) and the dimensions of the aorta ascendens (3mensio: 31.9 mm ± 4.2 vs. Sectra IDS7: 31.3 mm ± 3.7, ICC 0.756 [0.69–0.80]) to a poor correlation in the assessment of more complex structures like the virtual aortic annulus (3mensio: 22.9 mm ± 2 vs. Sectra IDS7: 24.7 mm ± 3, ICC 0.462 95% CI [0.17–0.63], which is crucial for sizing and the final determination of valve size. Further data is displayed in Table 1. Mean difference of the calculated diameter of the virtual aortic annulus averages 2.4 ± 2 mm. Considering 3mensio measurements as a reference, the varying calculated diameter results in different valve sizes in 47.1% of the cases predominantly due to oversizing (Table 2).

**Table 2:** Practical clinical impact of workflow on valve size selection.

Mean difference of	2.4 ± 2 (Mean ± SD)
calculated diameter (mm)	2 [1–3.4] (Median [IQR])
Different valve size based on	155 (47.1)
calculated diameter (%)
Oversizing (%)	135 (87.1)
Undersizing (%)	20 (12.9)

*Over- and undersizing are estimated considering 3mensio measurements as reference*.

## Discussion

Non-invasive imaging is a very powerful tool and may determine patient eligibility, the access site, and device selection, and helps to identify the best angiographic view for valve delivery (2). Even though, as a strength of this study, we have highly trained experts in both routinely used workflows, the interrater reliability between workflows varied significantly, especially in the assessment of the virtual aortic annulus where MSCT is defined as the gold standard tool for evaluation. Quantitative assessment requires accurate identification of the hinge points of the right and non-coronary cusps to create the virtual annular plane. This can be done manually (in case of Sectra IDS7) or on a software-based facilitated workflow (in case of 3mensio). Although no reference standard for this measurement has been approved, considering which of the two measurements is more correct, a software-based approach may provide a more accurate assessment by minimizing subjectivity. In a cohort of 105 patients, automated 3mensio software showed equally good reproducibility as manual measurement (3). The same applies to the 3mensio three-dimensional computed tomography (3D-CT) reconstruction tool with regard to accuracy and reproducibility (4). Furthermore, Foldyna et al. observed a significantly faster evaluation with semi-automatic rather than with manual segmentation of pre-interventional MSCT (5) with comparable exactness. In contrast, our results hint at the impact of workflows used in pre-interventional analysis and reveal a poor correlation in the assessment of more complex structures between different workflows despite extensively trained operators. Therefore, workflows have a relevant impact on correct valve sizing and the choice of device highlighting the limited reproducibility between different workflows. We, therefore, recommend harmonization of the routinely used workflows by interprofessional communication and training. Moreover, studies are evolving, which evaluate the feasibility of AI models and algorithms implemented in analysis software even for small cardiac structures, to detect moderate to high-grade coronary stenosis (6, 7). In the future, it might be promising to validate and standardize AI algorithms to overcome discrepancies in the measurement of complex structures and choose the prothesis with the best hemodynamic and prognostic outcome in patients with aortic valve stenosis scheduled for TAVR.

## Author Contributions

All authors listed have made a substantial, direct, and intellectual contribution to the work and approved it for publication.

## Funding

This study was supported by the Deutsche Forschungsgemeinschaft (DFG, German Research Foundation) – Grant No. 397484323.

## Conflict of Interest

VV and TZ have received consulting fees, travel expenses, or study honorariums from Medtronic and Edwards Lifesciences outside of this work. The remaining authors declare that the research was conducted in the absence of any commercial or financial relationships that could be construed as a potential conflict of interest.

## Publisher's Note

All claims expressed in this article are solely those of the authors and do not necessarily represent those of their affiliated organizations, or those of the publisher, the editors and the reviewers. Any product that may be evaluated in this article, or claim that may be made by its manufacturer, is not guaranteed or endorsed by the publisher.

## References

[1] Achenbach S Delgado V Hausleiter J Schoenhagen P Min JK Leipsic JA. expert consensus document on computed tomography imaging before transcatheter aortic valve implantation (TAVI)/transcatheter aortic valve replacement (TAVR). J Cardiovasc Comput Tomogr. (2012) 6:366–80. 10.1016/j.jcct.2012.11.002 23217460

[2] Otto CM Kumbhani DJ Alexander KP Calhoon JH Desai MY Kaul S. 2017 ACC expert consensus decision pathway for transcatheter aortic valve replacement in the management of adults with aortic stenosis: a report of the american college of cardiology task force on clinical expert consensus documents. J Am Coll Cardiol. (2017) 69:1313–46. 10.1016/j.jacc.2016.12.006 28063810

[3] Watanabe Y Morice MC Bouvier E Leong T Hayashida K Lefèvre T. Automated 3-dimensional aortic annular assessment by multidetector computed tomography in transcatheter aortic valve implantation. JACC Cardiovasc Interv. (2013) 6:955–64. 10.1016/j.jcin.2013.05.008 23954060

[4] Stortecky S Heg D Gloekler S Wenaweser P Windecker S Buellesfeld L. Accuracy and reproducibility of aortic annulus sizing using a dedicated three-dimensional computed tomography reconstruction tool in patients evaluated for transcatheter aortic valve replacement. EuroIntervention. (2014) 10:339–46. 10.4244/EIJV10I3A59 24273249

[5] Foldyna B Jungert C Luecke C von Aspern K Boehmer-Lasthaus S Rueth EM. CT evaluation prior to transapical aortic valve replacement: semi-automatic versus manual image segmentation. Int J Cardiovasc Imaging. (2015) 31:1233–42. 10.1007/s10554-015-0662-6 25893746

[6] Jonas RA Barkovich E Choi AD Griffin WF Riess J Marques H. The effect of scan and patient parameters on the diagnostic performance of AI for detecting coronary stenosis on coronary CT angiography. Clin Imaging. (2022) 84:149–58. 10.1016/j.clinimag.2022.01.016 35217284

[7] Infante T Cavaliere C Punzo B Grimaldi V Salvatore M Napoli C. Radiogenomics and artificial intelligence approaches applied to cardiac computed tomography angiography and cardiac magnetic resonance for precision medicine in coronary heart disease: a systematic review. Circ Cardiovasc Imaging. (2021) 14:1133–46. 10.1161/CIRCIMAGING.121.013025 34915726

